# Does the use of benzodiazepine influence the effectiveness of lifestyle intervention? Secondary analysis from the Sarcopenia and Physical fRailty iN older people strategies (SPRINTT) trial

**DOI:** 10.1093/ageing/afag257

**Published:** 2026-09-02

**Authors:** Elena Levati, Maria Beatrice Zazzara, Daniele Giuli, Emanuele Marzetti, Anna Picca, Riccardo Calvani, Matteo Tosato, Francesco Landi, Roberto Bernabei, Graziano Onder

**Affiliations:** Fondazione Policlinico Universitario “Agostino Gemelli” IRCCS, Rome 00168, Italy; Department of Aging, Orthopaedics and Rheumatological Sciences, Università Cattolica del Sacro Cuore, 00168 Rome, Italy; Fondazione Policlinico Universitario “Agostino Gemelli” IRCCS, Rome 00168, Italy; Department of Aging, Orthopaedics and Rheumatological Sciences, Università Cattolica del Sacro Cuore, 00168 Rome, Italy; École polytechnique fédérale de Lausanne, Lausanne, Switzerland; Fondazione Policlinico Universitario “Agostino Gemelli” IRCCS, Rome 00168, Italy; Department of Aging, Orthopaedics and Rheumatological Sciences, Università Cattolica del Sacro Cuore, 00168 Rome, Italy; Department of Medicine and Surgery, LUM University, Casamassima, Italy; Department of Aging, Orthopaedics and Rheumatological Sciences, Università Cattolica del Sacro Cuore, 00168 Rome, Italy; Fondazione Policlinico Universitario “Agostino Gemelli” IRCCS, Rome 00168, Italy; Fondazione Policlinico Universitario “Agostino Gemelli” IRCCS, Rome 00168, Italy; Department of Aging, Orthopaedics and Rheumatological Sciences, Università Cattolica del Sacro Cuore, 00168 Rome, Italy; Italialongeva, Rome, Italy; Fondazione Policlinico Universitario “Agostino Gemelli” IRCCS, Rome 00168, Italy; Department of Aging, Orthopaedics and Rheumatological Sciences, Università Cattolica del Sacro Cuore, 00168 Rome, Italy

**Keywords:** benzodiazepines, mobility disability, lifestyle intervention, exercise, older people

## Abstract

**Purpose:**

Benzodiazepine use is prevalent among older adults despite its recognised negative effects, including increased risk of falls, fractures and decline in functional status. Lifestyle interventions can preserve physical function and reduce the risk of disability. This study investigates whether benzodiazepine use reduces the effectiveness of lifestyle-based interventions on mobility disability.

**Methods:**

We performed a secondary analysis of the ‘Sarcopenia and Physical fRailty IN older people: multi-componenT Treatment strategies’ trial. The lifestyle intervention consisted of physical exercise and nutritional counselling. Benzodiazepine use was assessed based on baseline or longitudinal exposure (defined as use at baseline plus use at two or more additional time points). Cox regression models were used to ascertain the association between benzodiazepine use and mobility disability incidence. A separate analysis was conducted among participants with a Short Physical Performance Battery (SPPB) score 3 to 7.

**Results:**

Among the 1506 participants included (mean age 78.9 years; 71.5% female; 753 intervention vs. 753 control), 211 (14.0%) reported benzodiazepine use at baseline. Overall, 55.0% of baseline benzodiazepine users and 43.6% of non-users developed mobility disability during the follow-up (*P* = .003). When baseline benzodiazepine use was considered, no significant effect associated with multicomponent intervention (MCI) was shown among non-users (Hazard Ratio (HR) = 0.86; 95% Confidence Interval (CI) 0.72–1.03) and users (HR = 1.04; CI 0.67–1.62). In the subgroup of participants with SPPB scores 3 to 7, a significant effect of the MCI was observed only among non-users (HR = 0.80, 95% CI 0.66–0.97), while no ‘association’ was detected among users (HR = 1.06; 95% CI 0.65–1.71, *P* for interaction = .035). When longitudinal benzodiazepine exposure was considered, similar results were observed. In particular, in the SPPB score 3 to 7 subgroup, the beneficial effect of the MCI was observed only among non-users (HR = 0.79; 95% CI 0.64–0.96), while no ‘association’ was detected among users (HR = 1.13; 95% CI 0.60–2.13, *P* for interaction = .035).

**Conclusion:**

In frail older adults, benzodiazepine use significantly blunts the effectiveness of lifestyle interventions for preventing mobility disability. These findings support a prudent approach to benzodiazepine use in frail older patients.

## Key Points

- **Question.** Does benzodiazepine use reduce the effectiveness of lifestyle-based interventions on mobility disability?
- **Findings.** In a randomised clinical (SPRINTT) trial, benzodiazepine use significantly blunts the effectiveness of lifestyle interventions based on physical activity and nutritional counselling for preventing mobility disability.
- **Meaning.** Based on the results of secondary analyses from SPRINTT trial, benzodiazepine should be prescribed prudently in frail older patients.

## Introduction

Benzodiazepines use is estimated to be about 3% in general population [1], and the use of these medications worldwide is increasing [2]. Prevalence is higher among adults aged 65 years or older: in European countries, benzodiazepines are prescribed to almost 15% of older patients [3] and more than 10% of women and 6% of men aged 65 to 80 years filled at least one benzodiazepine prescription over a 1-year period [4]. The use of benzodiazepines in older adults is strongly discouraged due to the well-established association with increased risk of functional and cognitive decline, delirium, falls, fractures and motor vehicle crashes [5]. Older adults have increased pharmacodynamic sensitivity and reduced clearance of benzodiazepines, which increase the risk of side effects, and use of these medications may expose to the risks of abuse, misuse and dependence. Criteria to assess potentially inappropriate medications, including the American Geriatrics Society Beers Criteria [6] and the European Geriatric Medicine Society STOPP Criteria [7], advise against benzodiazepine use for generalised anxiety disorder and insomnia in older adults, and placed benzodiazepines on the list of medications that should be avoided in patients over 65 years of age. This concern may be even more relevant in frail older adults, in whom the potential negative effects of benzodiazepine use may be amplified, making this population particularly vulnerable to benzodiazepine-related functional decline [8, 9].

Lifestyle interventions, particularly those incorporating exercise programmes, have shown to improve physical function and preserve independence in older adults [10]. Evidence from large trials showed how lifestyle interventions including exercise and multicomponent programmes can prevent mobility disability and slow functional decline. In addition, physical exercise has been shown to mitigate several conditions, including depression, anxiety and insomnia. The Lifestyle Interventions and Independence for Elders Study (LIFE) [11] demonstrated that a long-term structured physical activity programme would reduce the risk of major mobility disability compared with a health education programme. The Sarcopenia and Physical fRailty IN older people: multi-componenT Treatment strategies (SPRINTT) project [12] showed that a multicomponent intervention (MCI)—based on physical activity with technological support and nutritional counselling—could reduce the risk of mobility disability in older adults with physical frailty and sarcopenia (PF&S) compared with a healthy ageing lifestyle educational programme. This effect seems stronger among frail or pre-frail older populations.

Despite the demonstrated benefits of exercise-based interventions, concomitant benzodiazepine use may attenuate their effectiveness in older adults. By enhancing GABAergic neurotransmission, benzodiazepines induce sedation, impair attention and slow psychomotor responses, potentially limiting the intensity and quality of participation in exercise sessions [13]. In addition, benzodiazepine-related deficits in executive function and motor learning may limit the neuromuscular adaptations that underlie improvements in gait, balance and mobility [13]. These effects may be particularly relevant in frail older adults, who are more susceptible to both the adverse effects of benzodiazepines and mobility decline. However, whether benzodiazepine use modifies the benefits of structured lifestyle interventions aimed at preventing mobility disability remains poorly understood.

Clarifying how benzodiazepine exposure interacts with lifestyle interventions may provide additional evidence to support prudent prescribing, particularly in frail populations. The aim of this study is to examine whether benzodiazepine use modifies the association of lifestyle-based interventions in preventing incident mobility disability among older adults, using data from the SPRINTT trial. The analysis focused specifically on the frailer subgroup of participants, defined by Short Physical Performance Battery (SPPB) [14] scores between 3 and 7, who demonstrated the greatest benefit from the life-style intervention in the SPRINTT trial.

## Methods

### The SPRINTT trial

This study analysed data from the SPRINTT trial, a single-blind, multicentre randomised controlled trial designed to compare the effectiveness of a MCI with a healthy ageing lifestyle education (HALE) programme to prevent mobility disability among initially non-disabled older adults with PF&S [10]. Both interventions were administered for up to 36 months, depending on when participants were recruited during the trial. The MCI comprised a combination of moderate intensity physical activity with technological support and nutritional counselling. Physical activity included aerobic, strength, flexibility and balance exercises. Adherence was ascertained by registering centre attendance and participant-completed diaries on frequency of home-based sessions. Instructors used the information to provide participants with personalised feedback on their performance goals to be reached as part of behavioural strategies to maximise adherence and remove possible disincentives. The nutritional component was designed to support the effects of the physical activity programme. The intervention involved individualised nutritional assessments and prescription of personalised dietary plans with two main targets: a daily energy intake of 25–30 kcal/kg bodyweight and a daily protein intake of at least 1.0–1.2 g/kg bodyweight. Adherence to nutritional prescriptions was ascertained through regular contacts with study staff during which participant feedback was collected and dietary plans reviewed. The healthy ageing lifestyle educational programme consisted of seminars and workshops on topics relevant to older adults (eg, vaccinations, chronic pain management, gastrointestinal and urological problems, technological devices, personal safety).

The study population consisted of adults aged 70 years or older with PF&S, characterised by a SPPB score between 3 and 9 points (scores range from 0 to 12, with lower scores indicating poorer physical function), low appendicular lean mass according to sex-specific cut-points recommended by the Foundation for the National Institutes of Health Sarcopenia Project [15] and the absence of mobility disability, operationalised as the ability to complete a 400-m walk test within 15 min, without sitting, pausing for more than 1 min, receiving assistance or using a walker. The exclusion criteria included self-reported walking disability, cognitive impairment (defined as a Mini-Mental State Examination score < 24/30), terminal illness, participation in a structured physical activity programme, contraindications to safely engage in trial activities as judged by local study doctors and plans to relocate outside the study area within the following 2 years.

### Study sample

Overall, 1519 participants (760 randomised to MCI, 759 to HALE) were recruited between January 2016 and November 2017 in 16 clinical sites across 11 European countries and followed for an average of 26.4 months. Among these, 1198 had an SPPB score 3 to 7. Further details of trial design, recruitment, primary outcome and participant flow through the study have been previously published [16, 17]. A summary description of the protocol is available on ClinicalTrials.gov (NCT02582138).

### Benzodiazepine use

Medications data were collected at baseline and every 6 months throughout the 36 month-study, by trained study researchers. Participants were asked to bring all prescription and non-prescription medications used in the 2 weeks before the assessment, in their original containers with medication label. Medications were classified according to the Anatomical Therapeutic Chemical (ATC) classification system by the World Health Organization Collaborating Centre for Drug Statistics Methodology [18]. Dosages, corresponding unit of measurement and route of administration were also collected.

Benzodiazepines were identified by the following ATC codes ‘N05BA’, ‘N05CD’, ‘N03AE’, ‘N05CF’.

Baseline exposure was defined based on benzodiazepine use at the baseline visit only. To evaluate longitudinal benzodiazepine exposure, analyses were restricted to participants with medication data available at baseline and at least two follow-up assessments. Participants were classified as continuous users if they reported benzodiazepine use at baseline and at least two follow-up assessments. Participants who never reported benzodiazepine use at any assessment were classified as never users. Participants with less than two follow-up assessments and those with intermittent or inconsistent benzodiazepine use patterns (i.e. reporting use at some but not all assessments) were excluded from these analyses to maximise the contrast between persistent exposure and non-exposure.

### Mobility disability

The primary outcome of the SPRINTT trial was mobility disability, operationalised as the inability to complete the 400 m walk test in <15 min without sitting, stopping for more than 1 min, requiring help or using a walker. The 400 m walk test was administered 3 months after randomisation and every 6 months thereafter. Mobility disability was considered to be present on the date participants failed the 400 m walk test, were unable to attempt the test or did not attempt the test. Participants who died before experiencing mobility disability were censored at the date of death.

### Covariates

Demographic variables were collected at baseline via a standardised questionnaire. These characteristics included age, sex and race. Clinical data included information on presence or absence of diseases, including CVD, hypertension, diabetes, history of hip fracture, arthritis, cancer, chronic lung disease, previous falls, stroke, anxiety and mood disorder. Cognitive and physical performance were assessed using the Mini Mental State Examination (MMSE), SPPB, body mass index and the 400-m walk test.

### Statistical analysis

Of the 1519 participants enrolled in the trial, 1506 (99.1%) had a complete medication assessment at the baseline evaluation, including 753/760 participants in the MCI group (99.1%) and 753/759 (99.2%) in the HALE group. Baseline characteristics of participants, stratified by benzodiazepine use, were described according to intervention arms as means and standard deviations (SDs) for continuous variables and counts (percentages) for categorical variables. Analyses of intervention effects were based on the intention-to-treat principle.

Cox proportional hazards models were used to examine the association between intervention group (MCI vs. HALE) and incident mobility disability according to benzodiazepine use status. Variables considered for adjustment were sex, age, study site and those significantly differing between benzodiazepine users and non-users in the whole sample, which included baseline 400 m speed, MMSE and total number of medications assessed at baseline assessment. Sex and study site were modelled as stratification factors, allowing for potentially non-proportional and complex effects on the baseline hazard function. The proportional hazards assumption was retained for all other covariates, including treatment arm, benzodiazepine use and their interaction. Such models were fitted both in the overall population and in the subgroup of participants with SPPB 3 to 7, analysing both baseline and longitudinal benzodiazepine exposure. In the longitudinal analysis, participants with less than two follow-up assessments and those with intermittent or inconsistent benzodiazepine use patterns (i.e. reporting use at some but not all assessments) were excluded from these analyses to maximise the contrast between persistent exposure and non-exposure. Subdistribution hazard models (Fine-Gray) were fitted to estimate sub-HRs for incident mobility disability, accounting for death as a competing risk.

To build on the results obtained by the models, the conditional average treatment effects (CATE) were identified and estimated. CATE is defined as the expected value of the difference between the potential outcomes under the treatment and control arms, conditional on benzodiazepine use. The identification was ensured under the standard assumption of positivity, consistency and conditional independence of the potential outcomes with the treatment, given the use of benzodiazepines and an SPPB score of 3 to 7. All analysis and figure generation were performed using R software (version 4.2.3).

## Results

### Sample characteristics

Among 1506 participants, mean age was 78.9 (SD 5.8) years and 1077 (71.5%) were women. Baseline characteristics of participants according to baseline benzodiazepine use and intervention arm (MCI vs. HALE) are reported in Table 1. Overall, 211 participants (14.0%) reported benzodiazepine use at baseline, whereas 142 participants (9.4% of the whole sample) reported benzodiazepine use at baseline and at least two additional follow-up assessments, being classified as continuous users. Lorazepam was the most used benzodiazepine (*n* = 48), followed by alprazolam (*n* = 30) and bromazepam (*n* = 28). Table 1 shows the distribution and frequency of benzodiazepine use among participants at baseline. The stratification of participants by benzodiazepine use did not introduce imbalance and the original randomisation remained preserved. No significant differences in the analysed variables were observed between MCI and HALE in both benzodiazepine user and non-user groups. Moreover, the balance achieved through randomisation was confirmed in analyses restricted to participants with baseline SPPB scores of 3 to 7 (Supplementary Table 1A in the Appendix), as well as in analyses selecting participants according to longitudinal benzodiazepine exposure (Supplementary Table 2A in the Appendix), where no evidence of imbalance between treatment groups emerged.

**Table 1:** Characteristics of participants according to baseline use on benzodiazepine and study arm*.*

Variable	Whole sample *N* = 1506	Benzodiazepine users *N* = 211 (14.0%)			Benzodiazepine no-users *N* = 1295 (86.0%)		
		HALE *N* = 99	MCI *N* = 112	*P*	HALE *N* = 654	MCI *N* = 641	*P*
**Demographics**							
Age (years)	78.9 (5.8)	79.1 (5.8)	79.7 (6.0)	.44	78.7 (5.7)	79.0 (5.9)	.38
Women	1077 (71.5%)	86 (86.9%)	101 (90.2%)	.59	450 (68.8%)	440 (68.6%)	.99
**Cognitive and physical measures**							
SPPB, score	6.7 (1.4)	6.7 (1.3)	6.5 (1.5)	.15	6.7 (1.4)	6.7 (1.3)	.47
Body mass index (kg/m^2^)	28.6 (5.7)	29.5 (6.1)	28.1 (5.3)	.22	28.5 (6.0)	28.7 (5.5)	.21
MMSE, score	27.9 (1.8)	28.0 (1.9)	28.0 (1.8)	.83	27.5 (1.8)	27.5 (1.9)	.80
400-m speed (m/s)	0.82 (0.21)	0.81 (0.18)	0.79 (0.19)	.60	0.83 (0.21)	0.83 (0.22)	.81
**Clinical characteristics**							
Osteoarthritis	1160 (77.0%)	80 (80.8%)	94 (83.9%)	.68	496 (75.8%)	490 (76.4%)	.85
Cancer	211 (14.0%)	15 (15.2%)	17 (15.2%)	.99	92 (14.1%)	87 (13.6%)	.86
Chronic lung disease	232 (15.4%)	18 (18.2%)	16 (14.3%)	.56	95 (14.5%)	103 (16.1%)	.49
Any falls	674 (44.6%)	44 (44.4%)	55 (49.1%)	.59	287 (43.9%)	288 (44.9%)	.75
Myocardial infarction	128 (8.5%)	5 (5.1%)	8 (7.1%)	.73	61 (9.3%)	54 (8.4%)	.64
Congestive heart failure	98 (6.5%)	6 (6.1%)	7 (6.3%)	.99	48 (7.3%)	37 (5.8%)	.31
Hypertension	999 (66.3%)	65 (65.7%)	81 (72.3%)	.37	418 (63.9%)	435 (67.9%)	.15
Hip fracture	91 (6.0%)	9 (9.1%)	9 (8.0%)	.98	35 (5.4%)	38 (5.9%)	.74
Stroke or brain haemorrhage	100 (6.6%)	3 (3.0%)	6 (5.4%)	.62	44 (6.7%)	47 (7.3%)	.75
Any cardiovascular disease	1077 (71.5%)	66 (66.7%)	85 (75.9%)	.18	457 (69.9%)	469 (73.2%)	.21
Diabetes mellitus	326 (21.6%)	13 (13.1%)	23 (20.5%)	.21	156 (23.9%)	134 (20.9%)	.23
Number of medications	6.0 (3.1)	7.1 (2.8)	7.8 (3.1)	.10	5.8 (3.1)	5.8 (3.1)	.98

Continuous variables are reported as means (standard deviations), and categorical variables as absolute frequencies (percentages).

In the overall study sample, including both participants with MCI and HALE groups, the mean age was similar between benzodiazepine users and non-users (79.4 vs. 78.8 years, *P* = .18). Compared with non-users, benzodiazepine users were more frequently women (88.6% vs. 68.7%, *P* < .001), had lower MMSE scores (27.6 vs. 28.0, *P* = .002), slower 400-m walking speed (0.80 vs. 0.83 m/s, *P* = .04) and used a higher number of medications (7.5 vs. 5.8 drugs, *P* < .001, Supplementary Table 3A in the Appendix).

#### Effect of benzodiazepine use on intervention effectiveness

**Figure 1:**
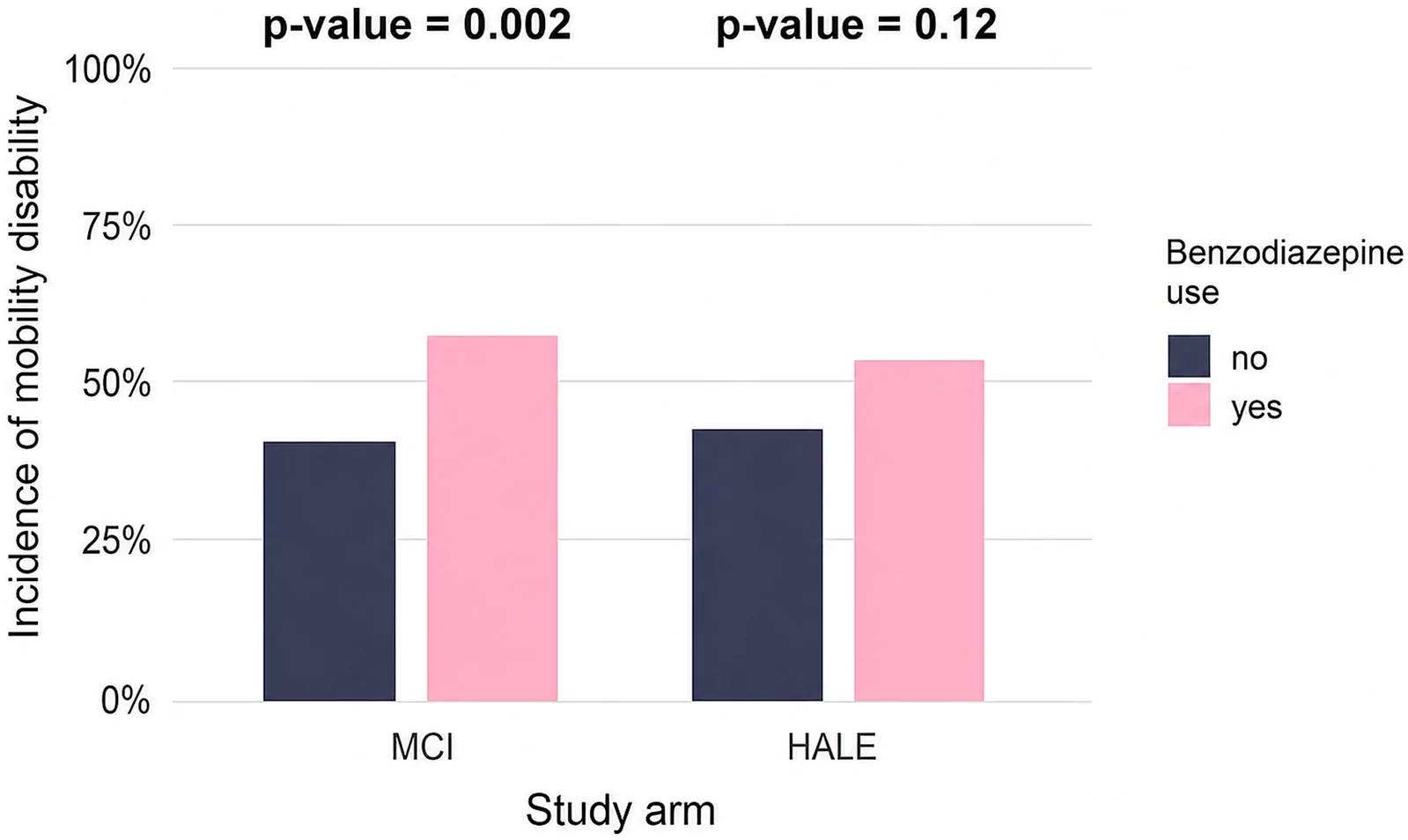
Incidence of mobility disability among study groups (MCI vs. HALE groups) according to baseline benzodiazepine use in the whole sample.

Overall, 55.0% of baseline benzodiazepine users and 42.2% of non-users developed mobility disability during the follow-up (*P* = .003). As shown in Figure 1, the higher incidence of mobility disability among baseline benzodiazepine users was consistent in both the MCI (56.3% vs. 39.9%, *P* = .002) and the HALE group (53.5% vs. 44.5%, *P* = .115). While the intervention effect (MCI vs. HALE) was not significant among benzodiazepine users (56.3% vs. 53.5%, *P* = .797) and among non-users (39.9% vs. 44.5%, *P* = .109).

Table 2 presents Cox proportional hazards regression models, adjusted for potential confounders, analysing the association between the study intervention and mobility disability, according to benzodiazepine use. When baseline benzodiazepine use was considered, no significant association was shown between MCI and mobility disability among both non-users (HR = 0.86; 95% CI 0.72–1.03) and users (HR = 1.04; CI 0.67–1.62). The interaction between intervention group and benzodiazepine use did not reach statistical significance (*P* = .201). In the subgroup of participants with SPPB scores 3 to 7, MCI was associated with a reduced probability of mobility disability only among non-users (HR = 0.80, 95% CI 0.66–0.97), while no association was detected among users (HR = 1.06; 95% CI 0.65–1.71), with a significant interaction between intervention arm and benzodiazepine use (*P* = .035).

**Table 2:** Incidence rates of mobility disability and hazard ratios comparing MCI vs HALE groups, stratified by benzodiazepine use (baseline and longitudinal exposure).

	Benzodiazepine use		No benzodiazepine use		*P* for interaction
	Incidence rate events/total sample (%)	Hazard ratio (95% CI)	Incidence rate events/total sample (%)	Hazard Ratio (95% CI)	
**Baseline exposure**					
**Total sample**	116/211 (55.0%)		547/1295 (42.2%)		
**MCI**	63/112 (56.3%)	1.04 (0.67–1.62)	256/641 (39.9%)	0.86 (0.72–1.03)	.201
**HALE**	53/99 (53.5%)		291/654 (44.5%)		
**Only SPPB 3–7**	108/168 (64.3%)		476/1028 (46.3%)		
**MCI**	59/90 (65.6%)	1.06 (0.65–1.71)	217/510 (42.5%)	0.80 (0.66–0.97)	.035
**HALE**	49/78 (62.8%)		259/518 (50.0%)		
**Longitudinal exposure** ^**a**^					
**Total sample**	79/142 (55.6%)		505/1222 (41.3%)		
**MCI**	41/75 (54.7%)	1.14 (0.64–2.03)	239/605 (39.5%)	0.84 (0.69–1.01)	.117
**HALE**	38/67 (56.7%)		266/617 (43.1%)		
**Only SPPB 3–7**	73/112 (65.2%)		441/970 (45.5%)		
**MCI**	38/58 (65.5%)	1.13 (0.60–2.13)	204/482 (42.3%)	0.79 (0.64–0.96)	.028
**HALE**	35/54 (64.8%)		237/488 (48.6%)		

Results are shown for the total sample and for participants with an SPPB score of 3 to 7. Models are adjusted for age, sex, study site, MMSE, 400-m speed and number of medications.

^a^Analyses were restricted to participants with medication data available at baseline and at least two follow-up assessments. Benzodiazepines use = use at baseline and at least two follow-up assessments (continuous use). No benzodiazepine use = no benzodiazepine use at any assessment (never use).

When longitudinal benzodiazepine exposure was considered, similar results were observed. In the total sample, the association between the study intervention and mobility disability remained non-significant among both continuous users (HR = 1.14; 95% CI 0.64–2.03) and never users (HR = 0.84; 95% CI 0.69–1.01, *P* for interaction = .023). In the SPPB score 3 to 7 subgroup, MCI was associated with a reduced probability of mobility disability only among never users (HR = 0.79; 95% CI 0.64–0.96), while no association was detected among continuous users (HR = 1.13; 95% CI 0.60–2.13, *P* for interaction = .035).

Sub-distribution hazard ratios were estimable only among non-users of benzodiazepines and were closely consistent with the corresponding hazard ratios from the cause-specific Cox model (Supplementary Table 4A in the Appendix). Among benzodiazepine users, no participant died prior to developing mobility disability, so death did not act as a competing risk in this subgroup, and only the standard hazard ratio is reported.

The analysis of effect heterogeneity further supports the results obtained by the models. The estimated CATE among non-users was −0.076 (95% CI −0.137 to −0.015), indicating that the intervention caused a 7.6% reduction in the incidence of mobility disability in the subgroup of participants with an SPPB score of 3–7. In contrast, the CATE among benzodiazepine users with an SPPB score of 3 to 7 was 0.027 (95% CI −0.118 to 0.173), indicating no evidence of an intervention benefit in this group. Similar results were obtained when benzodiazepine use was considered longitudinally (CATE = 0.007, 95% CI −0.17 to 0.184 for continuous users; CATE = −0.068, 95% CI −0.131 to −0.006 for never users).

## Discussion

The present study shows that benzodiazepine use attenuates the beneficial effects of a multicomponent lifestyle intervention in older adults with physical frailty. Notably, attenuation of the intervention effect was more pronounced in the frailer subgroup with SPPB scores of 3–7, where the interaction between benzodiazepine use and MCI arm reached statistical significance. This finding is consistent with the original SPRINTT trial, in which the intervention based on physical activity and nutritional counselling reduced the risk of incident mobility disability and improved physical performance primarily among participants with SPPB scores of 3–7. This observation is further supported by results from the LIFE study, where the hazard of incident mobility disability was significantly reduced by physical activity only among participants with SPPB scores below 8 [10]. Therefore, benzodiazepine use may be particularly relevant in this subgroup, potentially limiting the functional benefits of lifestyle interventions in those most likely to benefit from them.

To our knowledge, this is the first analysis from a clinical trial aiming to investigate the potential interaction of benzodiazepine use on the effect of lifestyle interventions. Several mechanisms may explain the attenuation of the intervention effect observed among benzodiazepine users. Benzodiazepine exposure may interfere with the mechanisms through which physical activity preserves mobility and independence [8, 9, 13]. This hypothesis is supported by observational evidence showing that benzodiazepine use is associated with impaired postural stability, slower reaction times and an increased risk of falls and fractures in older adults [19]. Furthermore, chronic benzodiazepine use has been linked to cognitive impairment, depressive symptoms and reduced motivation [20, 21], factors that may contribute to frailty progression and compromise participation in and responsiveness to lifestyle interventions. These effects may be particularly detrimental in vulnerable populations, such as older adults with physical frailty, prefrailty or mild cognitive impairment, in whom deficits in balance, coordination and cognitive function may limit the ability to fully benefit from exercise-based programmes.

Despite their well-documented adverse effects and inclusion in lists of potentially inappropriate medications, benzodiazepines remain widely prescribed in the older population [1–4]. Barriers to changing benzodiazepine prescribing patterns include the lack of scheduled medication reviews, under-recognition of adverse effects, a perceived favourable risk–benefit balance, limited visit time, concerns about jeopardising the physician–patient relationship and reluctance to question prescribing decisions made by other clinicians [22]. Even when physicians recognise that continued use may be unnecessary or inappropriate, discontinuation can be challenging because of withdrawal symptoms, rebound insomnia and anxiety.

Pharmacological and non-pharmacological alternatives for the management of insomnia and anxiety have been shown to be effective and should be prioritised in older adults [23]. Physical exercise itself may represent a relevant non-pharmacological strategy for improving insomnia and anxiety symptoms, potentially reducing reliance on benzodiazepines [10]. In this context, the US Food and Drug Administration highlights the risk of physical dependence and withdrawal symptoms associated with benzodiazepine use and emphasises the importance of limiting both dosage and duration to the minimum necessary to achieve the desired clinical effect [24]. Given the risk of withdrawal symptoms and clinical destabilisation, reducing or discontinuing these medications requires caution. To address this issue, the American Society of Addiction Medicine, in collaboration with nine other medical societies, developed guidance to support clinicians in benzodiazepine discontinuation [25], which suggest to gradually taper their dosage under appropriate medical supervision.

Several limitations merit consideration. First, evaluating benzodiazepine use was not the primary objective of the trial; prescription indications as well as medication continuation or discontinuation were managed by participants’ clinicians rather than being protocolised within the study. Second, only participants with complete medication inventory were included. The number of participants using benzodiazepines was relatively small, which limited statistical power and precluded more specific analyses (e.g. by dose, type of benzodiazepines, duration or indication). Third, because benzodiazepine exposure was not randomised, residual confounding by indication and disease severity cannot be entirely excluded despite multivariable adjustment and stratification. Finally, medication exposure was assessed at scheduled visits, which may have introduced misclassification of intermittent use and limited our capacity to model short-term changes in therapy.

In conclusion, this secondary analysis shows that benzodiazepine use among pre-frail and frail older adults reduces the effectiveness of a multicomponent lifestyle intervention aimed at reducing the incidence of mobility disability. Although assessment of benzodiazepine use was not specific to the SPRINTT trial, the available evidence supports the hypothesis that these medications may negatively affect outcomes closely related to mobility, physical performance and disability, which are central endpoints of lifestyle interventions. Further studies are needed to investigate the specific mechanisms by which benzodiazepine use may affect physical performance and responsiveness to lifestyle interventions.

Given the high prevalence of benzodiazepine use in older adults and their known adverse effects, careful medication review and deprescribing strategies should be integrated into lifestyle-based prevention programmes. Physicians should evaluate the appropriateness of benzodiazepine therapy, consider gradual tapering when feasible and prioritise non-pharmacological approaches for managing insomnia and anxiety. Overall, these results highlight the importance of coordinating pharmacological and lifestyle interventions in geriatric care to maximise the functional benefits of exercise and support healthy ageing.

## Supplementary Material

aa-26-0898-File003_afag257
