# Supplementary material for: Does the use of benzodiazepine influence the effectiveness of lifestyle intervention? Secondary analysis from the Sarcopenia and Physical fRailty iN older people strategies (SPRINTT) trial
Source: Age Ageing. 2026 Sep 2;55(8):afag257. doi: 10.1093/ageing/afag257 (PMC13537444; doi:10.1093/ageing/afag257)
Supplement: Supplementary file 1 [file aa-26-0898-file003_afag257.docx]

**APPENDIX**

**Table 1A.** Characteristics of participants with SPPB between 3 and 7 according to baseline use on benzodiazepine and study arm.

| **Variable** | **Whole sample N = 1,196** | **Benzodiazepine users N = 168** | | | **Benzodiazepine no-users N = 1,028** | | |
| --- | --- | --- | --- | --- | --- | --- | --- |
|  |  | **HALE N = 78** | **MCI N = 90** | **p** | **HALE**  **N = 518** | **MCI**  **N = 510** | **p** |
| **Demographics** | | | | | | | |
| Age (Years) | 79.2 (5.8) | 79.4 (6.1) | 80.2 (6.0) | 0.34 | 79.1 (5.7) | 79.1 (5.9) | 0.93 |
| Women | 852 (71.2%) | 69 (88.5%) | 82 (91.1%) | 0.76 | 353 (68.1%) | 348 (68.2%) | 0.99 |
| **Cognitive and physical measures** | | | | | | | |
| SPPB, score | 6.2 (1.1) | 6.3 (1.1) | 6.0 (1.2) | 0.10 | 6.7 (1.4) | 6.8 (1.3) | 0.58 |
| Body Mass Index (kg/m^2^) | 28.72 (5.67) | 29.23 (5.97) | 28.04 (5.45) | 0.18 | 28.52 (5.94) | 28.74 (5.52) | 0.50 |
| MMSE, score | 27.9 (1.8) | 27.4 (1.8) | 27.4 (1.9) | 0.90 | 28.1 (1.8) | 28.0 (1.8) | 0.54 |
| 400-m speed (m/s) | 0.79 (0.21) | 0.77 (0.17) | 0.76 (0.19) | 0.73 | 0.79 (0.21) | 0.80 (0.21) | 0.42 |
| **Clinical characteristics** | | | | | | | |
| Osteoarthritis | 923 (77.2%) | 64 (82.1%) | 76 (84.4%) | 0.84 | 396 (76.4%) | 387 (75.9%) | 0.89 |
| Cancer | 161 (13.5%) | 10 (12.8%) | 12 (13.3%) | 0.99 | 72 (13.9%) | 67 (13.1%) | 0.79 |
| Chronic lung disease | 186 (15.6%) | 11 (14.1%) | 13 (14.4%) | 0.99 | 76 (14.7%) | 86 (16.9%) | 0.38 |
| Any falls | 550 (46.0%) | 34 (43.6%) | 47 (52.2%) | 0.34 | 235 (45.4%) | 234 (45.9%) | 0.92 |
| Myocardial infarction | 96 (8.0%) | 4 (5.1%) | 6 (6.7%) | 0.93 | 46 (8.9%) | 40 (7.8%) | 0.63 |
| Congestive heart failure | 85 (7.1%) | 6 (7.7%) | 7 (7.8%) | 0.99 | 39 (7.5%) | 33 (6.5%) | 0.59 |
| Hypertension | 803 (67.1%) | 52 (66.7%) | 65 (72.2%) | 0.54 | 340 (65.6%) | 346 (67.8%) | 0.49 |
| Hip fracture | 69 (5.8%) | 8 (10.3%) | 6 (6.7%) | 0.58 | 26 (5.0%) | 29 (5.7%) | 0.74 |
| Stroke or brain  haemorrhage | 86 (7.2%) | 2 (2.6%) | 5 (5.6%) | 0.56 | 39 (7.5%) | 40 (7.8%) | 0.94 |
| Any cardiovascular  disease | 863 (72.2%) | 53 (67.9%) | 69 (76.7%) | 0.28 | 370 (71.4%) | 371 (72.7%) | 0.69 |
| Diabetes mellitus | 270 (22.6%) | 10 (12.8%) | 19 (21.1%) | 0.23 | 129 (24.9%) | 112 (22.0%) | 0.30 |
| Number of medications | 6.0 (3.1) | 7.1 (2.8) | 7.6 (3.0) | 0.26 | 5.8 (3.2) | 5.9 (3.1) | 0.74 |

MCI=Multicomponent intervention; HALE=Healthy Aging Lifestyle Education

SPPB=Short Physical Performance Battery; MMSE=Mini Mental State Examination

**Table 2A.** Characteristics of participants according to longitudinal use on benzodiazepine and study arm.

| **Variable** | **Whole sample N = 1,506** | **Benzodiazepine users N = 142** | | | **Benzodiazepine no-users N = 1,222** | | |
| --- | --- | --- | --- | --- | --- | --- | --- |
|  |  | **HALE** N = 67 | **MCI** N = 75 | **p** | **HALE**  N = 617 | **MCI**  N = 605 | **p** |
| **Demographics** | | | | | | | |
| Age (Years) | 78.9 (5.8) | 79.5 (6.1) | 79.2 (5.8) | 0.81 | 78.6 (5.8) | 78.9 (5.9) | 0.39 |
| Women | 1,077 (71.5%) | 58 (86.6%) | 68 (90.7%) | 0.61 | 421 (68.2%) | 414 (68.4%) | 0.99 |
| **Cognitive and physical measures** | | | | | | | |
| SPPB, score | 6.7 (1.4) | 6.6 (1.4) | 6.7 (1.5) | 0.91 | 6.7 (1.4) | 6.7 (1.3) | 0.77 |
| Body Mass Index (kg/m^2^) | 28.6 (5.7) | 29.02 (5.87) | 28.35 (5.32) | 0.48 | 28.60 (5.99) | 28.65 (5.44) | 0.86 |
| MMSE, score | 27.9 (1.8) | 27.6 (1.9) | 27.8 (1.8) | 0.54 | 28.0 (1.9) | 28.0 (1.8) | 0.21 |
| 400-m speed (m/s) | 0.82 (0.21) | 0.81 (0.19) | 0.80 (0.20) | 0.72 | 0.83 (0.21) | 0.82 (0.22) | 0.88 |
| **Clinical characteristics** | | | | | | | |
| Osteoarthritis | 1,160 (77.0%) | 57 (85.1%) | 67 (89.3%) | 0.61 | 468 (75.9%) | 461 (76.2%) | 0.94 |
| Cancer | 211 (14.0%) | 11 (16.4%) | 9 (12.0%) | 0.61 | 89 (14.4%) | 83 (13.7%) | 0.79 |
| Chronic lung disease | 232 (15.4%) | 12 (17.9%) | 13 (17.3%) | 0.99 | 87 (14.1%) | 95 (15.7%) | 0.48 |
| Any falls | 674 (44.6%) | 28 (41.8%) | 35 (46.7%) | 0.68 | 269 (43.6%) | 270 (44.6%) | 0.76 |
| Myocardial infarction | 128 (8.5%) | 4 (6.0%) | 4 (5.3%) | 0.99 | 57 (9.2%) | 52 (8.6%) | 0.77 |
| Congestive heart failure | 98 (6.5%) | 3 (4.5%) | 4 (5.3%) | 0.99 | 43 (7.0%) | 35 (5.8%) | 0.47 |
| Hypertension | 999 (66.3%) | 45 (67.2%) | 54 (72.0%) | 0.66 | 391 (63.4%) | 413 (68.3%) | 0.08 |
| Hip fracture | 91 (6.0%) | 8 (11.9%) | 4 (5.3%) | 0.27 | 33 (5.3%) | 38 (6.3%) | 0.57 |
| Stroke or brain  haemorrhage | 100 (6.6%) | 2 (3.0%) | 3 (4.0%) | 0.99 | 42 (6.8%) | 46 (7.6%) | 0.67 |
| Any cardiovascular  disease | 1,077 (71.5%) | 46 (68.7%) | 58 (77.3%) | 0.33 | 428 (69.4%) | 445 (73.6%) | 0.12 |
| Diabetes mellitus | 326 (21.6%) | 9 (13.4%) | 12 (16.0%) | 0.85 | 143 (23.2%) | 126 (20.8%) | 0.36 |
| Number of medications | 6.0 (3.1) | 7.2 (2.9) | 7.7 (3.2) | 0.31 | 5.7 (3.1) | 5.7 (3.0) | 0.95 |

MCI=Multicomponent intervention; HALE=Healthy Aging Lifestyle Education

SPPB=Short Physical Performance Battery; MMSE=Mini Mental State Examination

**Table 3A.** Characteristics of participants according to baseline use on benzodiazepine in the whole sample.

| **Variable** | **Whole sample N = 1,506** | **Benzodiazepine users N = 211** | **Benzodiazepine no-users N = 1,295** | **p-value** |
| --- | --- | --- | --- | --- |
| **Demographics** | | | | |
| Age (Years) | 78.9 (5.8) | 79.4 (5.9) | 78.8 (5.8) | 0.18 |
| Women | 1,077 (71.5%) | 87 (88.6%) | 890 (68.7%) | <0.001 |
| **Cognitive and physical measures** | | | | |
| SPPB, score | 6.7 (1.4) | 6.6 (1.4) | 6.7 (1.4) | 0.27 |
| Body Mass Index (kg/m^2^) | 28.6 (5.7) | 28.7 (5.7) | 28.6 (5.7) | 0.78 |
| MMSE, score | 27.9 (1.8) | 27.6 (1.8) | 28.0 (1.8) | 0.002 |
| 400m speed (m/s) | 0.82 (0.21) | 0.80 (0.19) | 0.83 (0.22) | 0.04 |
| **Clinical characteristics** | | | | |
| Osteoarthritis | 1160 (77.0%) | 174 (82.5%) | 986 (76.1%) | 0.15 |
| Cancer | 211 (14.0%) | 32 (15.2%) | 179 (13.8%) | 0.81 |
| Chronic lung disease | 232 (15.4%) | 34 (16.1%) | 198 (15.3%) | 0.96 |
| Any falls | 674 (44.6%) | 99 (46.9%) | 575 (44.4%) | 0.69 |
| Myocardial infarction | 128 (8.5%) | 13 (6.2%) | 115 (8.9%) | 0.43 |
| Congestive heart failure | 98 (6.5%) | 13 (6.2%) | 85 (6.6%) | 0.99 |
| Hypertension | 999 (66.3%) | 146 (69.2%) | 853 (65.9%) | 0.57 |
| Hip fracture | 91 (6.0%) | 18 (8.5%) | 73 (5.6%) | 0.29 |
| Stroke or brain haemorrhage | 100 (6.6%) | 9 (4.3%) | 91 (7.0%) | 0.35 |
| Any cardiovascular disease | 1,077 (71.5%) | 151 (71.6%) | 926 (71.5%) | 0.99 |
| Diabetes mellitus | 326 (21.6%) | 36 (17.1%) | 290 (22.4%) | 0.23 |
| Number of medications | 6.0 (3.1) | 7.5 (3.0) | 5.77 (3.1) | <0.001 |

SPPB=Short Physical Performance Battery; MMSE=Mini Mental State Examination

**Table 4A.** Hazard ratios for mobility disability, and subdistribution hazard ratios (accounting for death as a competing risk) comparing Multicomponent Intervention (MCI) vs. Healthy Aging Lifestyle Education (HALE) groups, stratified by benzodiazepine use (baseline and longitudinal exposure).

|  | **Benzodiazepine use** | | **No benzodiazepine use** | |
| --- | --- | --- | --- | --- |
|  | Hazard Ratio  (95% CI) | Sub- Hazard Ratio  (95% CI) | Hazard Ratio  (95% CI) | Sub- Hazard Ratio  (95% CI) |
| **Baseline exposure** | | | | |
| **Total sample** |  |  |  |  |
| **MCI** | 1.04 (0.67–1.62) | —a | 0.86 (0.72–1.03) | 0.86 (0.73– 1.02) |
| **HALE** |  |  |  |  |
| **Only SPPB 3-7** |  |  |  |  |
| **MCI** | 1.06 (0.65–1.71) | —a | 0.80 (0.66–0.97) | 0.81 (0.67– 0.97) |
| **HALE** |  |  |  |  |
| **Longitudinal exposure*** | | | | |
| **Total sample** |  |  |  |  |
| **MCI** | 1.14 (0.64–2.03) | —a | 0.84 (0.69–1.01) | 0.85 (0.71– 1.01) |
| **HALE** |  |  |  |  |
| **Only SPPB 3-7** |  |  |  |  |
| **MCI** | 1.13 (0.60–2.13) | —a | 0.79 (0.64–0.96) | 0.80 (0.66– 0.97) |
| **HALE** |  |  |  |  |

MCI=Multicomponent intervention

HALE=Healthy Aging Lifestyle Education

CI=Confidence Interval

* *analyses restricted to participants with medication data available at baseline and at least two follow-up assessments. Benzodiazepines use = use at baseline and at least two follow-up assessments (continuous use). No benzodiazepine use = no benzodiazepine use at any assessments (never use).*

ᵃ Not estimable: no competing event (death before mobility disability) observed in this subgroup.
